# Pulse-SILAC and Interactomics Reveal Distinct DDB1-CUL4–Associated Factors, Cellular Functions, and Protein Substrates

**DOI:** 10.1016/j.mcpro.2023.100644

**Published:** 2023-09-07

**Authors:** Jennifer Raisch, Marie-Line Dubois, Marika Groleau, Dominique Lévesque, Thomas Burger, Carla-Marie Jurkovic, Romain Brailly, Gwendoline Marbach, Alyson McKenna, Catherine Barrette, Pierre-Étienne Jacques, François-Michel Boisvert

**Affiliations:** 1 Département d’Immunologie et de Biologie cellulaire, faculté de médecine et des sciences de la santé, Université de Sherbrooke, Sherbrooke, Québec, Canada; 2 Département de biologie, faculté des Sciences, Université de Sherbrooke, Sherbrooke, Québec, Canada; 3 CNRS, INSERM, Université Grenoble Alpes, Grenoble, France

**Keywords:** pulse-SILAC, BioID, interactome, DDB1, cullin4, DCAFs, ubiquitination

## Abstract

Cullin-RING finger ligases represent the largest family of ubiquitin ligases. They are responsible for the ubiquitination of ∼20% of cellular proteins degraded through the proteasome, by catalyzing the transfer of E2-loaded ubiquitin to a substrate. Seven cullins are described in vertebrates. Among them, cullin 4 (CUL4) associates with DNA damage–binding protein 1 (DDB1) to form the CUL4–DDB1 ubiquitin ligase complex, which is involved in protein ubiquitination and in the regulation of many cellular processes. Substrate recognition adaptors named DDB1/CUL4-associated factors (DCAFs) mediate the specificity of CUL4-DDB1 and have a short structural motif of approximately forty amino acids terminating in tryptophan (W)-aspartic acid (D) dipeptide, called the WD40 domain. Using different approaches (bioinformatics/structural analyses), independent studies suggested that at least sixty WD40–containing proteins could act as adaptors for the DDB1/CUL4 complex. To better define this association and classification, the interaction of each DCAFs with DDB1 was determined, and new partners and potential substrates were identified. Using BioID and affinity purification–mass spectrometry approaches, we demonstrated that seven WD40 proteins can be considered DCAFs with a high confidence level. Identifying protein interactions does not always lead to identifying protein substrates for E3-ubiquitin ligases, so we measured changes in protein stability or degradation by pulse-stable isotope labeling with amino acids in cell culture to identify changes in protein degradation, following the expression of each DCAF. In conclusion, these results provide new insights into the roles of DCAFs in regulating the activity of the DDB1–CUL4 complex, in protein targeting, and characterized the cellular processes involved.

## Highlights

• Analyzing DDB1 and DCAFs interactomes to define true DDB1/Cul4-associated factors.
• Proteomic investigation of DCAFs degradome to identify protein substrates of CRL4.
• DCAFbase allows easy visualization and browsing of each DCAFs results.

## Graphical Abstract

**Figure:**
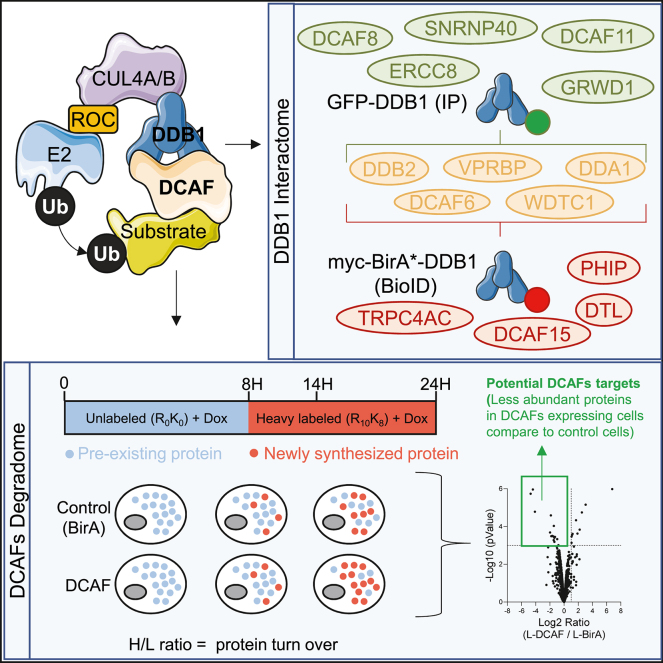

## In Brief

Previous analyses revealed that DDB1 interacts with up to sixty DCAFs shown to serve as substrate receptors for the CRL4 complex. Several DCAFs have yet to be confirmed and their targets remain to be identified. We identified partners and substrates for each DCAFs by interactomics and pulse-SILAC labeling. We created an interface allowing visualization, where each result is accessible for all the DCAFs. The characterization of 58 DCAFs gives us a new insight into targets of the DDB1–CRL4 complex.

Ubiquitin-mediated proteolysis is a key mechanism regulating almost every biological process (1). During ubiquitination, ubiquitin is first activated through a covalent thiol–ester bond to the E1 activating ubiquitin enzyme. Then, ubiquitin is transferred to a reactive cysteine on an E2-conjugating enzyme by transesterification, and finally, an E3 ubiquitin ligase covalently attaches ubiquitin to lysine on a substrate protein (2). So far, two families of E3 ligases have been described that contain either a homologous to E6-AP C terminus domain or the RING domain (3, 4, 5, 6, 7, 8). The homologous to E6-AP C terminus domain involves a mandatory thioester intermediate with a cysteine in the active site of the E3 (9), whereas the RING domain E3 enzymes will mediate the direct transfer of ubiquitin from the E2 to the substrate protein (10, 11). The cullin-RING ubiquitin ligases (CRLs) family uses one of the cullins as a central scaffold to bridge an E2 enzyme to the substrate to bring specificity in the substrate recognition (12). In vertebrates, CRLs represent one of the largest families of ubiquitin ligases and are responsible for approximately 20% of protein degradation through the proteasome (13). Among the seven cullins found in vertebrates, CUL4A and CUL4B (encoded by two genes) regulate numerous key functions in cells, including DNA repair, replication, cell cycle progression, or tumorigenesis (14). Despite sharing approximately 80% of protein sequence homology, CUL4A and CUL4B differ in several ways. Indeed, harboring a nuclear localization signal, CUL4B localizes mostly in the nucleus, while CUL4A is found predominantly in the cytoplasm (14). CRL complexes formed by CUL4A and CUL4B are very similar and structurally indistinguishable (15). Moreover, CUL4A and CUL4B are likely redundant and can compensate each other’s loss of expression, as no perturbation in phenotype and cell cycle progression was observed in *Cul4a*^−/−^ mouse embryonic fibroblasts (16).

Cullin 4 (CUL4) associates with DNA damage–binding protein 1 (DDB1) as an adaptor to form the DDB1–CUL4 (also called CRL4) ubiquitin ligase complex (17). DDB1 contains three WD40-like β-propeller domains (BPA, BPB, BPC), of which BPB interacts with the N-terminal part of CUL4A or CUL4B (18, 19). Moreover, DDB1 interacts with WD40 domain–containing proteins through an H-box domain (20, 21). WD40 is a short structural motif of around forty amino acids ending in tryptophan (W)-aspartic acid (D) dipeptide (17) found in 262 to 349 proteins in human (22, 23). Previous proteomics, bioinformatics and structural analyses revealed that DDB1 could potentially interact with up to sixty WD40 proteins through an additional consensus tandem domain DXXXR/KXWDXR/K (D: Aspartic acid; R/K: Arginine or Lysine; W: Tryptophan) (24, 25). These DWDs proteins (DDB1-binding WD40 proteins), named DDB1-CUL4–associated factors (DCAFs), were shown to serve as substrate receptors for the CRL4 complex (17, 24). However, despite being shown to interact with DDB1 experimentally, DCAF15, DCAF16, DCAF17, DET1, and DDA1 lack the WD40 motif or other interacting domains (26).

The specificity of the DDB1–CUL4 complex is thus mainly mediated by proteins belonging to the DCAF family, allowing the CRL4 complex to target specific proteins. The CRL4 complex was first identified for its role in genomic stability and DNA damage repair (15). Indeed, DDB2 was the first identified DCAF and is involved in nucleotide excision repair by recognizing UV-induced DNA damage sites. DDB1-DDB2 heterodimers are recruited to DNA damage, which serves as a platform to recruit other proteins involved in DNA repair and lead to ubiquination of substrates such as histones, destabilizing nucleosomes to allow DNA repair (27, 28). CSA (also named ERCC8), another WD40 protein and substrate receptor for the CRL4 complex, also plays a key role in transcription-coupled DNA repair (29). Moreover, mutations in DDB2 and CSA genes lead to DNA repair defects and to the development of *Xenoderma Pigmentosum* and Cockayne syndromes, respectively, highlighting the essential role of these DCAFs in cellular functions (29).

Previous studies identifying protein substrates for ubiquitination by the CRL4 complex revealed 25 potential targets, but not all were associated with chromatin, suggesting a diversity of cellular functions (12). With up to sixty substrates receptors, the CRL4 complex could potentially modulate several uncharacterized biological processes. However, several DCAFs have yet to be confirmed experimentally and their targets remain to be identified. To investigate the unknown roles of the CRL4 complex and to determine the proteins targeted for ubiquitination, we identified potential partners and substrates for each DCAFs by interactomics and pulse-stable isotope labeling with amino acids in cell culture (SILAC) labeling. Our results provide a better understanding of the potential regulatory role of the CRL4 complex in human cells.

## Experimental Procedures

### Generation of pcDNA-DEST53-BirA∗, pGLAP1-myc-BirA∗, and pGLAP1-GFP Constructions

The DCAFs and DDB1 coding sequences were obtained by PCR using complementary DNA (cDNA) generated from HeLa and HEK293 cells RNA. The sequences were then cloned into the pDONR221 vector (Thermo Fisher Scientific) by gateway cloning *via* BP reaction and into pGLAP1-myc-BirA∗ or pGLAP1-GFP vector *via* LR reaction, according to the manufacturer’s instructions (Thermo Fisher Scientific). VprBP was amplified from pCMV-myc-VprBP plasmids (a gift from Dr Eric A. Cohen) then cloned into pENTR11 vector using NotI and EcoRI restriction enzymes before being cloned into pDEST53-myc-BirA∗ plasmids *via* LR reaction, according to the manufacturer’s instructions (Thermo Fisher Scientific). All plasmids were sequenced *via* the Genome Sequencing and Genotyping Platform (Université Laval). Oligonucleotides used for amplification were obtained from Integrated DNA Technologies and are listed in Supplemental Table S1.

### Generation of Inducible Stable Cell Lines and Western Blot

The stable U2OS cell lines expressing myc-BirA∗-DCAF, myc-BirA∗-DDB1, and GFP-DDB1 were generated using the Flp-In T-Rex system (Thermo Fisher Scientific) using respectively pGLAP1-myc-BirA∗-DCAF, pGLAP1-myc-BirA∗-DDB1, and pGLAP1-GFP-DDB1 constructions. U2OS Flp-In-Rex (U2OS-FT) cells were maintained at 37 °C, 5% CO_2_ in Dulbecco’s Modified Eagle Medium (Thermo Fisher Scientific), supplemented with 10% FB essence (Wisent, St John’s), 50 U/ml penicillin/streptomycin, and 10 mM Hepes. U2OS-FT cells were transfected using Lipofectamine LTX (Thermo Fisher Scientific) during 48 h in 6 cm Petri dishes with 4.5 μg of Flp-Recombinase expression vector pOG44 (Thermo Fisher Scientific) and 500 ng of plasmid DNA. Transfected cells were selected for 2 weeks with hygromycin (50 μg/ml, Thermo Fisher Scientific) and blasticidin (10 μg/ml, Wisent). The expression of the cDNA was achieved by adding 10 μg/ml doxycycline (Clontech Laboratories, Mountain View) in the medium for 24 h or 48 h (or not as control). Cells were lysed directly in *Laemmli* sample buffer, and the extracted proteins were resolved by SDS-PAGE prior to being transferred to a nitrocellulose membrane. Immunoblotting was performed with a BirA∗ antibody (Novus Biologicals #6C4c7, 1:1000 dilution) or GFP (Santa Cruz Sc-9996, 1:1000 dilution). To inhibit cullins, we treated cells 24 h with 10 μM MLN4924 (Cell signaling 85923S).

### Immunofluorescence

Cells were seeded onto glass coverslips, grown for 24 h then treated with doxycycline for another 24 h. Cells were rinsed twice with ice-cold PBS, fixed with methanol for 20 min at −20 °C, and washed four times with cold PBS. The cells were incubated with 10% goat serum in PBS for 20 min and were then incubated in primary antibodies overnight for BirA∗ (Novus Biologicals #6C4c7, 1:400 dilution) in 10% goat serum in PBS. After two PBS washes, the cells were incubated with AlexaFluor 568 goat anti-mouse (Invitrogen # A-11004, 1:800) at room temperature for 1 h. Following two more PBS washes, we stained the nuclei with 4',6-diamidino-2-phenylindole (1 μg/μl) for 10 min at room temperature, washed them twice with PBS and mounted them with Immuno Mount (Thermo Fisher Scientific). GFP-DDB1–expressing cells were fixed with 4% paraformaldehyde, and we stained the nuclei with 4',6-diamidino-2-phenylindole (1 μg/μl) for 10 min at room temperature, washed them twice with PBS, and mounted them with Immuno Mount (Thermo Fisher Scientific).

### Coimmunoprecipitation

We grew cells in 15 cm Petri dishes until 50% confluency. After 48 h of doxycycline treatment, GFP-DDB1–expressing cells were harvested by scraping in PBS and lysed in a nondenaturing lysis buffer (50 mM Tris ph7.4, 150 nM NaCl, 1% Triton X-100, 1 mM PMSF) supplemented with EDTA-free Protease Inhibitor Mixture inhibitors (Roche) and nuclease (Sino Biological Inc). Total cell extracts were sonicated four times on ice with a Sonic Dismembrator Model 120 (Thermo Fisher Scientific) at 20% during 20 to 30 s, then incubated 30 min at 4 °C with rotation, and centrifuged 10 min at 12,000*g*. One to two micrograms of total protein was incubated with 20 μl of GFP-trap agarose beads from Chromatek for 3 h at 4 °C. We then washed the beads in lysis buffer and transferred them to a low-bind tube prior to processing for mass spectrometry (MS).

### Quantitative Real-Time PCR

The myc-BirA∗-DCAFs–expressing U2OS were incubated for 24 h with doxycycline to induce DCAFs expression. Total RNA was extracted using RNeasy RNA isolation kit (Qiagen). The concentration was measured by NanoDrop (Thermo Fisher Scientific). cDNAs were obtained using SuperScript II Reverse Transcriptase (Thermo Fisher Scientific). The expression of potential targets was analyzed by real-time quantitative PCR by the RNomics Platform (Université de Sherbrooke, https://rnomics.med.usherbrooke.ca/services/qrt-pcr), using LightCycler 96 (Roche Applied Science). The oligonucleotides used for the amplification of the tested genes were obtained from Integrated DNA Technologies (Supplemental Table S1). *MRPL19, PUM1, and YWHAZ* were used as reference genes.

### Proximity Labeling Assay

We grew cells in 15 cm Petri dishes until 50% confluency, treated them with doxycycline for 24 h, then added biotin (50 μM, Sigma-Aldrich) to the medium for another 24 h. We harvested the cells by scraping in PBS and lysed them in 1 ml of denaturing lysis buffer (50 mM Tris–HCl, pH 7.5, 150 mM NaCl, 1.5 mM MgCl 2, 0.1% SDS, 1% IGEPAL CA-630 [Sigma-Aldrich]) supplemented with 1 mM PMSF, 0.4% sodium deoxycholate, 1 mM DTT, 1 mM EDTA, and the EDTA-free Protease Inhibitor Mixture inhibitor. The cell lysates were incubated on a rotator for 20 min at 4 °C and sonicated on ice with a Sonic Dismembrator Model 120 (Thermo Fisher Scientific) at 30% amplitude three times for 10 s. We added SDS (0.4% final concentration) to samples before a second incubation on a rotator for 20 min at 4 °C. We centrifuged the cell lysates 20 min at 4 °C at 2400*g* and put aside the supernatant. We then quantified proteins using a Pierce Bicinchoninic Acid Protein Assay kit (Thermo Fisher Scientific). High-performance streptavidin beads (Cytiva; #17511301) were added and incubated overnight at 4 °C under rotation with 1 to 2 mg of total protein then washed once with 1 ml of wash buffer (50 mM Tris–HCl, pH 7.5, 2% SDS) and three times with 1 ml lysis buffer. After a transfer into low-bind tubes, beads were washed five times in 20 mM ammonium bicarbonate buffer (in MS-grade water). All washes were performed by rotating the beads for 5 min at 4 °C and subsequently centrifuging at 800*g* for 5 min at 4 °C before removing the supernatant.

We carried out the reduction step by incubating the beads at 60 °C for 30 min under agitation (1250 rpm) with 100 μl of 20 mM ammonium bicarbonate buffer supplemented with DTT (10 mM final concentration). Samples were alkylated by adding 100 μl of 20 mM ammonium bicarbonate buffer supplemented with chloroacetamide (15 mM final concentration) (Sigma #C0267-100G) for 1 h at room temperature, protected from the light. Chloroacetamide was then quenched by adding DTT to reach a final concentration of 15 mM during 10 min upon agitation (1250 rpm). Beads were incubated overnight at 37 °C with 1 μg of Pierce trypsin protease MS-Grade (Thermo Fisher Scientific, #PI90058). All buffers used for reduction, alkylation, and digestion were prepared in MS-grade water. Trypsin was stopped by acidifying with a final concentration of 1% formic acid (FA) (Thermo Fisher Scientific, #A11750). After centrifugation at 800*g* for 5 min, we put aside the supernatant and incubated beads with 200 μl of buffer containing 60% acetonitrile (ACN) (Thermo Fisher Scientific, #A9554) and 0.1% FA. We then centrifuged them again and removed the supernatant to combine it with that obtained previously. In order to concentrate the samples, we achieved complete drying with a centrifugal evaporator at 60 °C (∼2 h) and resuspended them in 30 μl of 0.1% TFA buffer (Thermo Fisher Scientific, #A11650). We purified the peptides with ZipTip 10 μl micropipette tips containing a C18 column (Thermo Fisher Scientific). Briefly, the ZipTip was first moistened with 10 μl of 100% ACN solution three times then equilibrated with 10 μl of 0.1% TFA buffer three times. Each peptide sample was passed on the balanced ZipTip by 10 up-and-downs of 10 μl of the sample. This step was performed three times to pass the entire sample on the column. We then washed the ZipTip with 10 μl of 0.1% TFA buffer three times. The elution of the peptides was performed in a new low-binding tube, ten times with a volume of 10 μl of 50% ACN and 0.1% FA buffer. We repeated this step three times to obtain a final volume of 30 μl. The peptides were then concentrated by centrifugal evaporator at 65 °C until complete drying, then resuspended in 25 μl of 1% FA buffer. Peptides were quantified using a NanoDrop spectrophotometer (Thermo Fisher Scientific) and read at an absorbance of 205 nm. We then transferred the peptides into a glass vial (Thermo Fisher Scientific) and stored it at −20 °C until the MS analysis.

### Pulse-Chase SILAC and Validation of Potential Substrates

Cells were grown until 70% of confluency. They were then incubated with doxycycline (10 μg/ml) in a R_0_K_0_ medium to induce myc-BirA∗-DCAF or myc-BirA∗ expression. After 8 h, we replaced the R_0_K_0_ medium with a R_10_K_8_ medium containing doxycycline for 16 h. We then harvested the samples in lysis buffer (8 M urea [Sigma #U5128–5 kg], Hepes 50 mM), and 50 μg of proteins were incubated and boiled for 2 min with DTT 5 mM. We then diluted the samples four times in 20 mM ammonium bicarbonate buffer, digested them by adding 1 μg Pierce MS-grade trypsin (Thermo Fisher Scientific), and incubated them overnight at 37 °C with shaking. Peptides were purified with ZipTip 100 μl micropipette tips containing a C18 column (Thermo Fisher Scientific), as previously described in the proximity labeling assay section, concentrated with a centrifugal evaporator at 65 °C until complete drying then resuspended in 25 μl of 1% FA buffer. Peptide concentration was determined using a NanoDrop spectrophotometer (Thermo Fisher Scientific) and read at an absorbance of 205 nm. We then transferred the peptides to a glass vial (Thermo Fisher Scientific) and stored them at −20 °C until the MS analysis. To validate potential substrates, cells were grown in 6-well plates until 70% of confluency and were then incubated with doxycycline (10 μg/ml) during 24 h to induce myc-BirA∗-DCAF or myc-BirA∗ expression and were then treated with MLN4924 (10 μM) or DMSO during 24 h. We then harvested samples in lysis buffer (8 M urea [Sigma #U5128–5 kg], Hepes 50 mM), and 50 μg of proteins were processed for MS analysis as described above.

### Mass Spectrometry

#### LC-MS/MS Analysis

After trypsin digestion, we separated the peptides using a Dionex Ultimate 3000 nanoHPLC system. A total of 1.5 μg of peptides in 1% (v/v) FA were loaded with a constant flow of 4 μl/min onto an Acclaim PepMap100 C18 column (0.3 mm id × 5 mm, Dionex Corporation). After trap enrichment, peptides were eluted onto an EasySpray PepMap C18 nano column (75 μm × 50 cm, Dionex Corporation) with a linear gradient of 5 to 35% solvent B (90% ACN with 0.1% FA) over 240 min with a constant flow of 200 nl/min. The HPLC system was coupled to an OrbiTrap QExactive mass spectrometer (Thermo Fisher Scientific) *via* an EasySpray source. The spray voltage was set to 2 kV and the temperature of the column to 40 °C. Full scan MS survey spectra (*m/z* 350–1600) in profile mode were acquired in the Orbitrap with a resolution of 70,000 after 1,000,000 ions accumulated. The ten most intense peptide ions from the preview scan in the Orbitrap were fragmented by collision-induced dissociation (normalized collision energy of 35% and resolution of 17,500) after 50,000 ions accumulated. Maximal filling times were 250 ms for the full scans and 60 ms for the tandem mass spectrometry (MS/MS) scans. We enabled the precursor ion charge state screening to reject all unassigned charge states, as well as singly, seven and eight charged species. The dynamic exclusion list was restricted to 500 entries at most, with a maximum retention period of 40 s and a relative mass window of 10 ppm. We enabled the lock mass option survey scans to improve mass accuracy. We retrieved the data using the Xcalibur software (version 4.3.73.11; https://www.thermofisher.com/order/catalog/product/OPTON-30965).

#### Protein Identification by MaxQuant Analysis

The raw files were analyzed using the MaxQuant software (version 1.6.7; https://www.maxquant.org/) and the UniProt human database (2020/03/21, 75,777 entries). The settings used for the MaxQuant analysis were: two miscleavages were allowed; fixed modification was carbamidomethylation on cysteine; enzyme was Trypsin (K/R not before P); variable modifications included in the analysis were methionine oxidation, protein N-terminal acetylation, and carbamylation (K and N-terminal, only for pulse-SILAC experiments). We used a mass tolerance of 7 ppm for precursor ions and a tolerance of 20 ppm for fragment ions and the following parameters were used: multiplicity of two SILAC media (R0K0, and R10K8), identification values “peptide-to-spectrum match false discovery rate,” “Protein FDR,” and “Site decoy fraction” of 0.05, minimum ratio count of 1 and the “Requantify” option was selected. Following the analysis, the results were filtered according to several parameters (see experimental design and statistical rationale section).

#### Experimental Design and Statistical Rationale

We conducted analyses of the interaction between DDB1 and DCAF by combining the proximity labeling assay and the coimmunoprecipitation approach coupled to SILAC-based quantitative MS. We compared two conditions: the control cell lines in light medium (R_0_K_0_) and a cell line expressing DDB1 fused to GFP or BirA∗ grown in heavy medium (R_10_K_8_). We used two methods in biological triplicates to increase evidence of interactions between DCAF and DDB1. To increase the confidence in the results obtained, we performed a proximity labeling assay in biological duplicates on DCAF-expressing cells to identify DDB1 and CRL members as interactors, using probabilistic scoring of affinity purification (SAINT score). To be considered as DCAF interactor, proteins should have a SAINT score over 0.7. We conducted the experiments in biological duplicates. Results of pulse-chase SILAC experiments are presented as Volcano plot with Prostar (see below), and the differential analysis of L intensities after myc-BirA∗-DDB2 and ERCC8 expression was performed using four independent biological replicates for each condition (and two replicates for the rest of the DCAFbase). Proteins with a log2 fold change (FC) ≤−1 (between DCAF-expressing cells and control cells) and which passed the FDR significancy threshold in Prostar (see below) were considered to be less abundant and to be potential substrates.

Statistical analyses were performed using Prostar software tools (30, 31), with the following parameters: filtering (contaminants, reverse, only identified by sites, minimum of two unique peptides; missing values (MVs): a maximum of 1 MV for partially observed conditions (termed partially observable value (POV) MVs) authorized as well as missing on the entire condition (MECs) when N = 4; no POVs when N = 2); median normalization; SLSA imputation on POVs and DetQuantile (1%) on MECs; Limma moderated *t* test, no cut-off on the FC and FDR with the following procedure: (1) the correct calibration of the raw *p*-value was visually assessed using a calibration plot; (2) no adjustment on the proportion of null hypothesis ($\pi_{0})$; (3) adjusted *p*-values were computed using Benjamini–Hochberg (original, *i.e.*, $\pi_{0}=1$) procedure; (4) for all the proteins with a raw *p*-value $\leq 10^{-3}$ (*i.e.*, $\log_{10}\left(p.val\right)\geq 3$), we verified that the adjusted *p*-values was ≤5%, as to guarantee an FDR control at risk 0.05 or lower; and we retained them as differentially abundant (*i.e.* the null hypothesis was rejected). For potential substrates validation, label-free quantification were used to analyzed protein abundancy and MVs (POVs and MECs) were imputed with a minimum value (DetQuantile (1%)).

## Results

### Expression of DCAF in Human Tissues

**Fig. 1:**
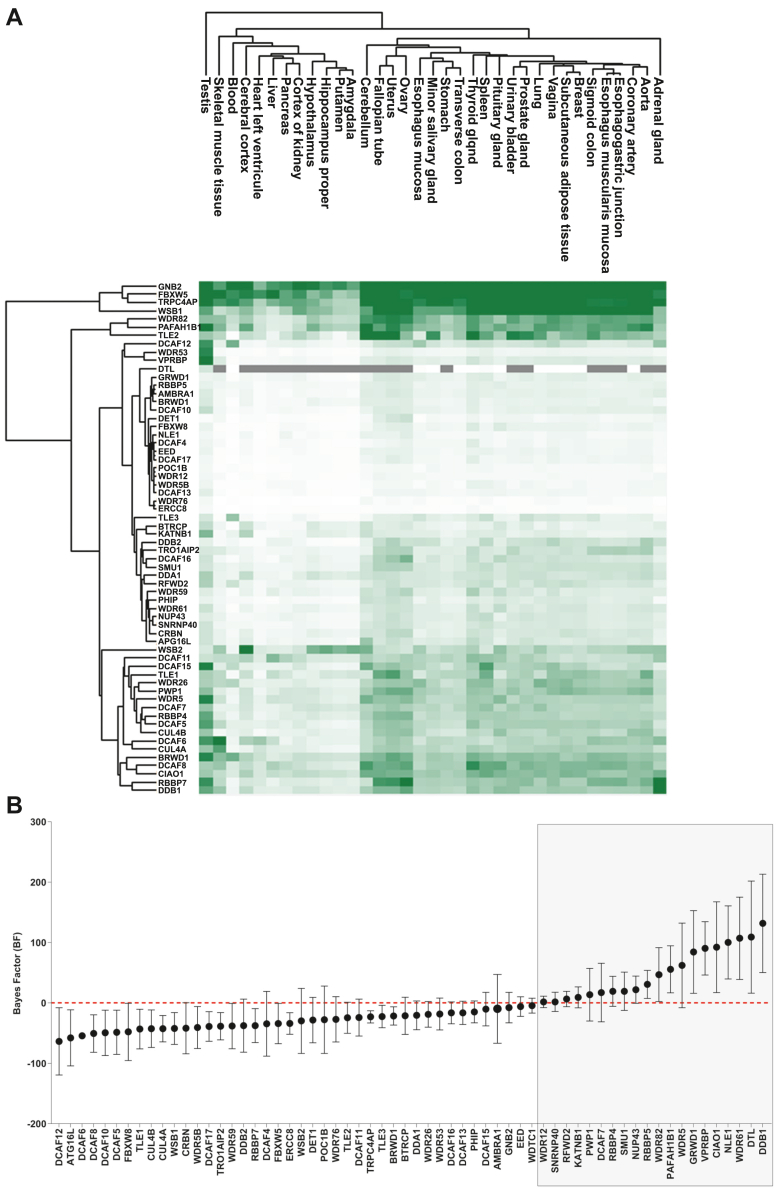
**DCAF expression in human tissue and essential properties for cell viability.***A*, hierarchical clustering heat map showing gene expression profiles of DCAFs in 35 tissues of healthy individuals from the Genotype-Tissue Expression project (GTex). Genes expressions were clustered using the Euclidian clustering method and appear with a gradient color with genes having a higher expression in *dark green*. The *gray region* represents genes with no expression observed. *B*, graph depicting essential properties of DCAFs for cell viability based on a calculated Bayes factor (BF) previously determined on five human cell lines. Genes with a BF over 0 are considered essential for cell viability. DCAFs considered essential appear in *red*. DCAF, DDB1-CUL4–associated factor.

DCAFs are characterized by the presence of the WD40 domain with an extended WDXR motif (17, 24, 25). Using different approaches, independent studies identified 59 proteins that could act as adaptors for the DDB1/CUL4 complex (24) (Supplemental Table S2). To determine the expressions of genes encoding these proteins, we analyzed RNA-Seq data from 35 tissues of healthy individuals from the Genotype-Tissue Expression project (32). All DCAFs were generally expressed in all the tissues analyzed with some variation in their expression, except for DTL (gene name: DCAF2), which was found in only 12 tissues and had a high level of expression in the testis (Fig. 1*A*). To define whether DCAFs harbor a pattern of expression in human tissues, transcripts per million were clustered using the Euclidian clustering method (Fig. 1*A*). Most DCAFs have a lower expression in muscle tissues, brain tissues, the gastrointestinal tract system (stomach, liver and pancreas), blood, and the left ventricle. Otherwise, the expression of DCAFs is generally high in the uterus, ovary, testis, spleen, and thyroid gland. To determine which DCAFs could be considered essential for cell viability, we ranked all the putative DCAFs based on a fitness CRISPR-Cas9 screen performed in five human cell lines (33). Based on a calculated Bayes factor (BF) measuring that the gene knockout results in a fitness defect in cell lines, 19 DCAFs (20%) were found to be essential with a BF over 0 (Fig. 1*B*). Moreover, with a BF of 131.7, DDB1 is the most essential gene tested, confirming its essential central role within the CRL4 complex. However, CUL4A and CUL4B presented a low BF, suggesting that one paralog can compensate for the absence of the other.

### Interaction of DCAFs With DDB1 CRL Complex

**Fig. 2:**
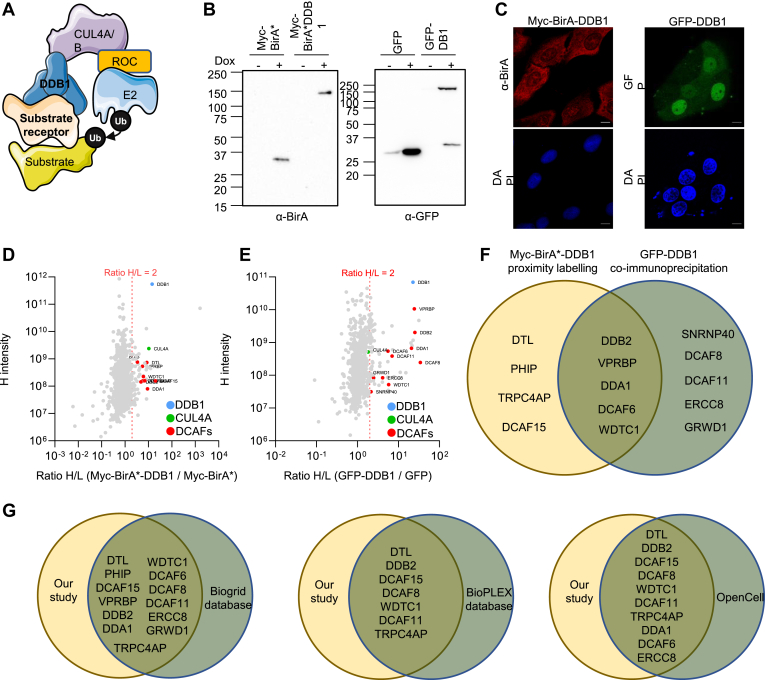
**DDB1-CUL4–associated factors interactome.***A*, schematic depicting DDB1–CRL4A/B E3 ligase complex. *B*, U2OS-FT stable cell lines expressing myc-BirA∗, myc-BirA∗-DDB1, GFP, and GFP-DDB1 were treated during 24 h with doxycycline (10 μM) to induce the expression of fused proteins. Cells were lysed and protein expression was determined anti-BirA∗ and anti-GFP antibodies. *C*, immunofluorescence using anti-BirA∗ antibody and autofluorescence of GFP in U2OS-FT stable cell lines expressing myc-BirA∗, myc-BirA∗-DDB1, GFP, and GFP-DDB1 treated during 24 h with doxycycline (10 μM). *D* and *E*, *dot plot* for all proteins detected in the myc-BirA∗-DDB1 proximity labeling assay and the GFP-DDB1 proximity labeling assay in SILAC condition. We defined a threshold of 2 to consider a protein as enriched in cells overexpressing DDB1 fused proteins (N = 3). DCAFs appear in *red*, CUL4A and DDB1 in *green*. *F*, venn diagram representing DCAFs found enriched in the myc-BirA∗-DDB1 proximity labeling assay and the GFP-DDB1 AP-MS. *G*, venn diagram showing DCAFs identified as DDB1 interactor in both BioID and AP-MS experiment compared to BioPlex, BioGRID, and OpenCell databases. AP-MS, affinity purification–mass spectrometry; CRL, Cullin-RING ubiquitin ligase; CUL4, cullin 4; DDB1, DNA damage–binding protein; SILAC, stable isotope labeling with amino acids in cell culture.

To identify which DCAFs could interact with the CRL4 complex (Fig. 2*A*), myc-BirA∗-DDB1 and GFP-DDB1 fused protein were expressed in U2OS Flp-In T-Rex inducible cell lines by adding doxycycline for 48 h. Immunoblotting confirmed the expression of the fused proteins (Fig. 2*B*) and immunofluorescence microscopy confirmed localization. Both GFP-DDB1 and myc-BirA∗-DDB1 were localized in the nucleus, with myc-BirA∗-DDB1 being also present in the cytoplasm (Fig. 2*C*), consistent with the known DDB1 localization (34) (Human Protein Atlas proteinatlas.org).

Potential DDB1 partners were analyzed by both proximity labeling assay and immunoprecipitation, followed by quantitative MS identification of proteins using SILAC. Stable cell lines expressing myc-BirA∗-DDB1 or GFP-DDB1 were grown in heavy medium (R_10_K_8_), while control cells, only expressing myc-BirA∗ or GFP, were grown in low medium (R_0_K_0_). Fourteen of the 59 annotated DCAFs were enriched in myc-BirA∗-DDB1 or GFP-DDB1 expressing cells compared to control, with a H/L ratio over 2 (Fig. 2, *D* and *E* and Supplemental Table S3). Moreover, five DCAFs were enriched in both experiments (DDB2, VprBP, DDA1, DCAF6, WDTC1). Otherwise, four were only enriched in myc-BirA∗-DDB1 proximity labeling experiments only (DTL, PHIP, TRPC4AC, DCAF15) and five were only enriched in GFP-DDB1 pulldown only (SNRNP40, DCAF8, DCAF11, ERCC8, GRWD1) (Fig. 2*F*). Interestingly, all of them except SNRNP40 were also found as DDB1 interactors in BioPlex (35), BioGRID (36), and OpenCell (37) protein interaction databases (Fig. 1*G*). DTL, DDB2, DCAF15, DCAF8, WDTC1, DCAF11, and TRPCA4P were found in DDB1 interactomes of the BioPlex, BioGRID, and OpenCell. Moreover, DDA1, DCAF6, and ERCC8 were identified as DDB1 partners in BioGRID and OpenCell databases, while PHIP, VprBP, and GRWD1 were only reported in the BioGRID database.

### DCAFs Interactome

Only identify a fraction (23%) of the suggested DCAFs were identified by using DDB1 as bait using two different approaches. While this could suggest no interaction between the remaining putative DCAFs and DDB1, it could also be that some DCAFs are not be expressed in U2OS cells or that the interactions occur under specific conditions, such as UV damage for DDB2 (15, 29). To confirm that the putative DCAFs are indeed DCAFs interacting with the CRL4 complex, we used a reciprocal approach. We proceeded to clone the cDNAs encoding all the DCAFs and managed to generate plasmids for 58 of the potential DCAFs, which we then validated for their expression by immunoblotting and localization by immunofluorescence microscopy. The cellular localization of the 58 potential DCAFs analyzed by immunofluorescence showed 31 (53%) in the cytoplasm, 15 (26%) in the nucleus, and 12 (21%) in both cellular compartments (Supplemental Table S4).

**Fig. 3:**
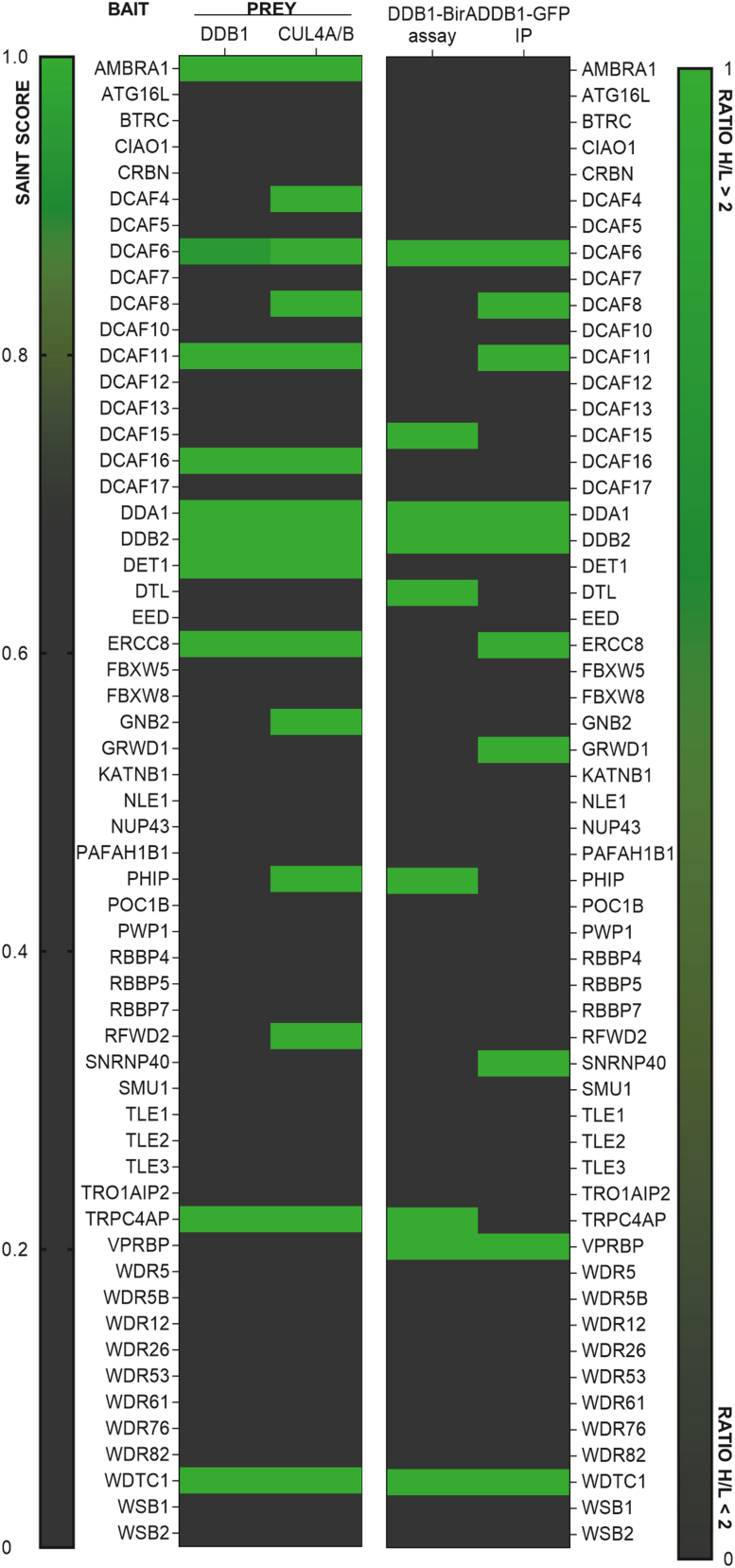
**DDB1-CUL4–associated factors interactome.** Heatmap depicting the results of 59 potential DCAFs from myc-BirA∗-DDB1 BioID (ratio H/L over 2) and GFP-DDB1 AP-MS (SAINT Score) experiments. Potential DCAFs identified as DDB1 interactors appear in *green*. Potential DCAFs considered as DCAFs with high confidence level appear in *red*. AP-MS, affinity purification–mass spectrometry; CUL4, cullin 4; DDB1, DNA damage–binding protein.

**Fig. 4:**
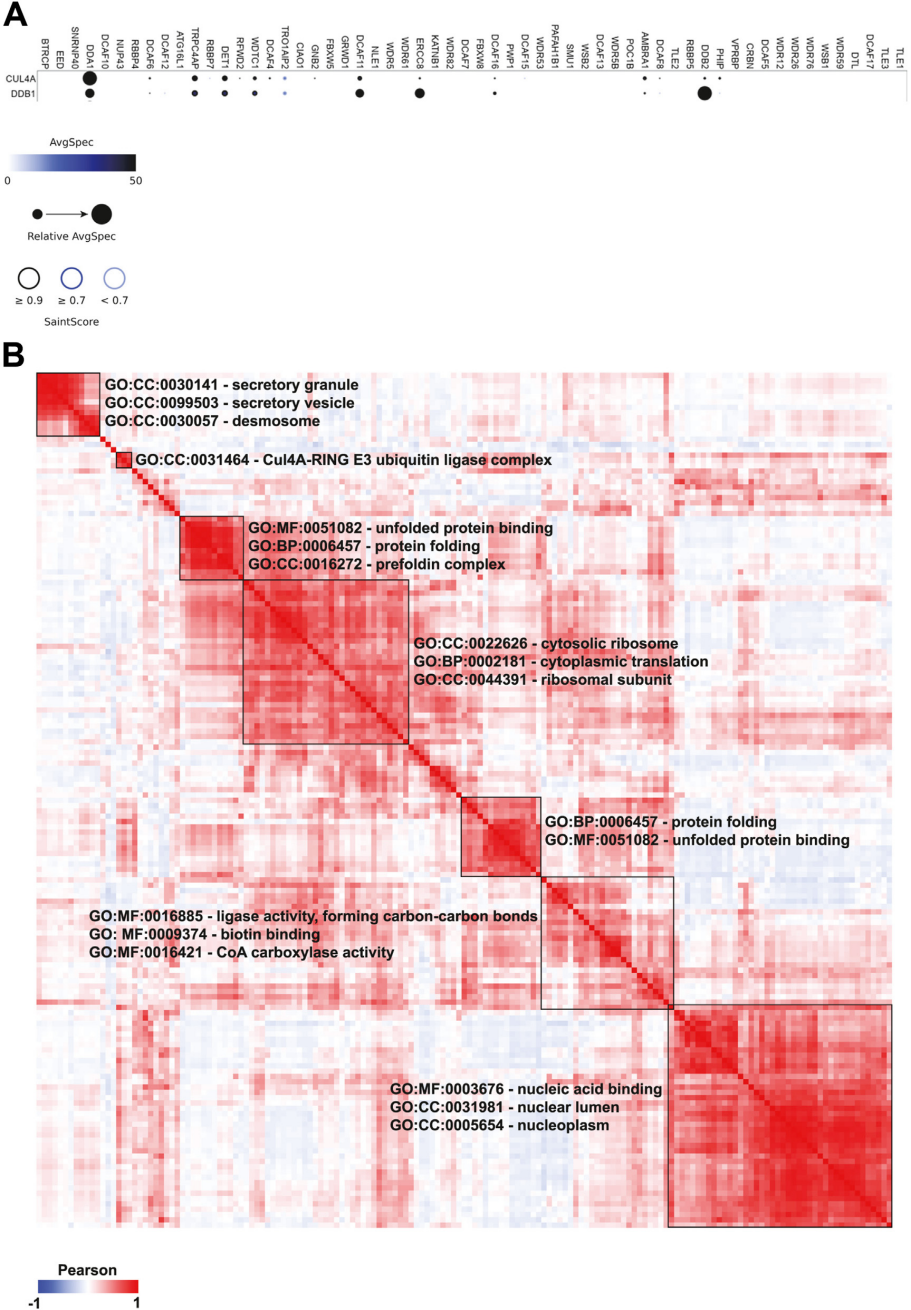
**Functional enrichment of DCAFs interacting proteins.***A*, *dot plot* of DDB1, CUL4A, COSP7A identified in DCAFs BioID experiments. The nodes’ color displays the average spectral count (N = 2), the node edge color corresponds to the SAINT Score, and the node size represents the relative abundancy of DDB1, CUL4A, and COSP7A across the 58 DCAFs compared. *B*, heatmap depicting Pearson’s correlation coefficients for interactors detected across the 58 DCAFs studied. Only interactors which were found in at least 20% of DCAFs BioID experiments were retained. Gene function enrichment results are depicted on the heatmap and were performed with g:profiler. CUL4, cullin 4; DCAF, DDB1-CUL4–associated factor; DDB1, DNA damage–binding protein.

To identify which DCAF could interact with the CRL4 complex, a proximity labeling assay (BioID) was performed on cell lines stably expressing each of the 58 different myc-BirA∗-DCAFs. DDB1 or CULA/B were identified as interactors (with a Saint Score between 0.7 and 1) in 15 of the 58 DCAFs tested (AMBRA1, DCAF4, DCAF6, DCAF8, DCAF11, DCAF16, DDA1, DDB2, DET1, ERCC8, GNB2, TRPC4AP, PHIP, RFWD2, WDTC1), of which ten were enriched for both DDB1 and CUL4A/B (AMBRA1, DCAF6, DACF11, DCAF16, DDA1, DDB2, DET1, ERCC8, TRPC4AP, WDTC1) (Figs. 3 and 4*A* and Supplemental Table S5).

To compare the interactome of DCAFs and define overlapping interactions, we calculated a global comparison based on MS/MS counts obtained in proximity labeling experiments and Pearson’s correlation coefficients for the interactome. The results are shown as a heat map including the correlation coefficients (Fig. 3). To be more accurate, the analysis was restricted to interacting proteins found in at least 20% of DCAFs, and we normalized the data by subtracting the MS/MS count of the corresponding control. We could observe seven clusters defined as proteins that could colocalize, be part of the same complex or be true interactors (Fig. 4*B*, Supplemental Table S6). Unsurprisingly, the pathway enrichment analysis of each cluster using GO terms (biological process, molecular function, and cellular components) revealed an enrichment of the CUL4A RING E3 ubiquitin ligase complex. Additionally, secretory granules, nuclear acid binding, protein folding, and biotin ligase/binding activity were also found to be enriched, although the biotin ligase activity is likely the result of the streptavidin enrichment of proteins. Altogether, these results highlighted seven DCAFs with high confidence levels based on their identification as DDB1 partners in both GFP-DDB1 affinity purification–mass spectrometry (AP-MS) or myc-BirA∗-DDB1 proximity labeling assay and myc-BirA∗-DCAF proximity labeling assay (DCAF6, DCAF11, DDA1, DDB2, TRPC4AC, ERCC8, WDTC1). We also identified ten other DCAFs with lower confidence levels because of their identification as DDB1 partners in only one of the reciprocal experiments (AMBRA1, DCAF4, DCAF15, DCAF16, DTL, GNB2, GRWD1, RFWD2, SNRNP40, VprBP) (Fig. 3). For some DCAFs such as VprBP, the low levels following expression could be due to instability and thus affect the identification of CUL4, DDB1, or interactors.

### Defining New Substrates of CUL4A/B–DDB1–DCAF Complex

**Fig. 5:**
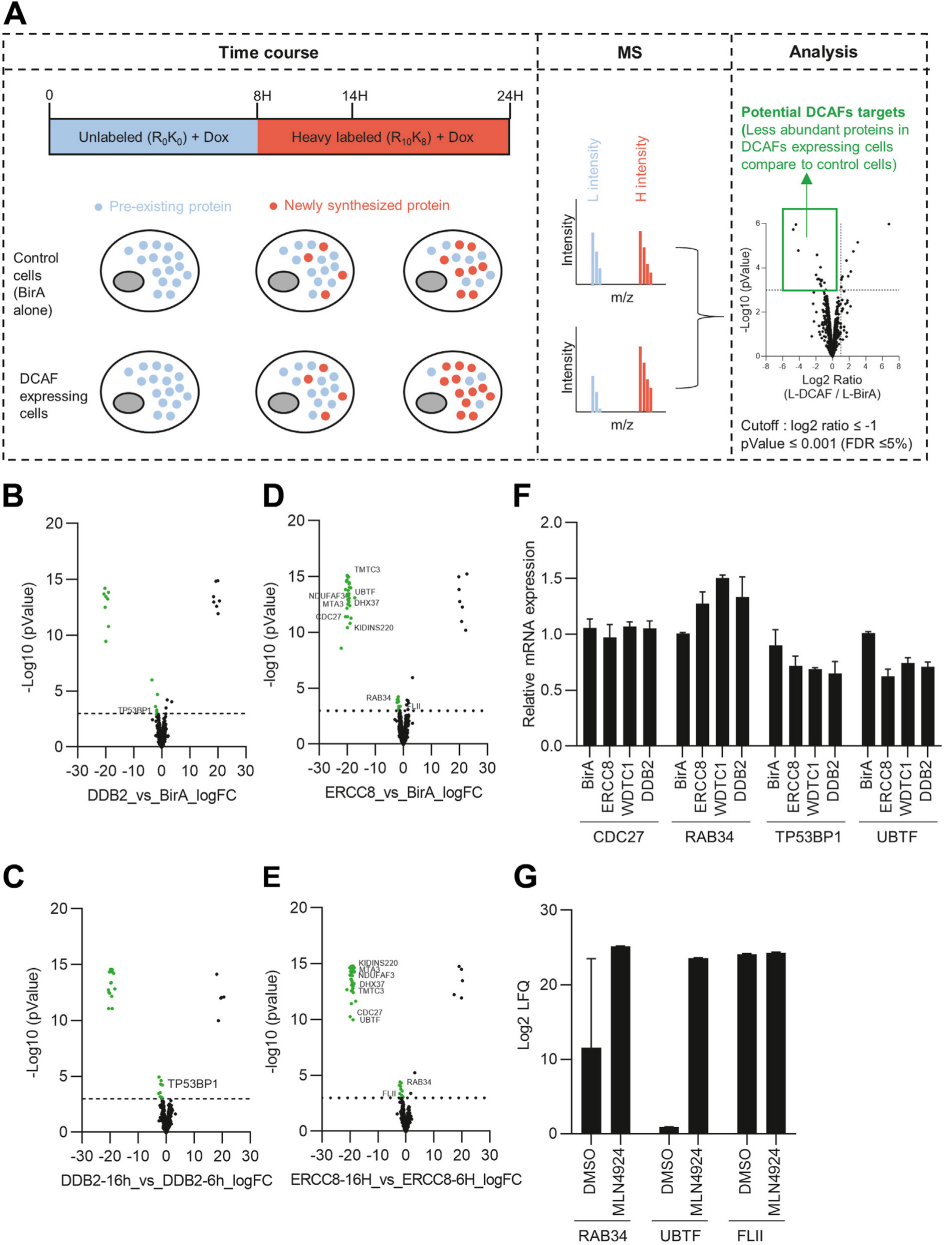
**Defining new substrates of CUL4A/B–DDB1–DCAF complex.***A*, schematic depicting pulse-SILAC experiments. Cells were cultured in a light medium (R_0_K_0_) during 8 h, then the medium was replaced for a heavy medium (R_10_K_8_) until 14 and 24 h. Cell lysates were processed for mass spectrometry analysis. We determined the decrease in protein level by calculating the ratio of protein between L-intensities in myc-BirA∗-DCAFs expressing cells compared to the L-intensities in control condition. *B* and *D*, *volcano plot* representing modulated protein abundancy from pulse-SILAC experiments in myc-BirA∗-DDB2 and myc-BirA∗-ERCC8 expressing cells compared to control myc-BirA∗ expressing cells 16 h after low medium replacement (N = 4). Proteins with a decreased abundancy appear in green (log2 ratio ≤1, *p*-value ≤ 0.001). *C* and *E*, *volcano plot* representing modulated protein abundancy overtime from pulse-SILAC experiments in myc-BirA∗-DDB2 and myc-BirA∗-ERCC8 expressing cells 16 h after low medium replacement compared to 6 h after low medium replacement (N = 4). Proteins with a decreased abundancy appear in *green* (log2 ratio ≤1, *p*-value ≤ 0.001). *F*, expression of potential targets of DDB2 and ERCC8 (TP53BP1, UBTF, RAB34, and CDC27) was assessed by semiquantitative PCR in myc-BirA∗-DDB2 and ERCC8 expressing U2OS cells after 24 h of doxycycline treatment (N = 2). *G*, graph representing protein abundancy of potential targets of ERCC8 (label-free quantification of RAB34, UBTF, and FLII) after cullins inhibitor treatment (MLN4924, 10 μM during 24 h) determined by mass spectrometry in myc-BirA∗-ERCC8 expressing U2OS cells. CUL4, cullin 4; DCAF, DDB1-CUL4–associated factor; DDB1, DNA damage–binding protein; SILAC, stable isotope labeling with amino acids in cell culture.

Identifying proteins interacting with DCAFs does not necessarily mean they are targeted for ubiquitination and degradation. Moreover, the interaction of a protein with a DCAF could result in its rapid degradation, preventing its identification using the previous BioID approach. Changes in protein stability could instead be used to identify proteins that DCAFs target for degradation by a more direct approach using an amino acid isotope pulse-chase experiment (38, 39). This MS-based approach has the advantage of measuring protein degradation without drugs or inhibitors (*e.g.*, cycloheximide), which can interfere with normal cellular activity and cell cycle progression. The method is based on the temporal incorporation of heavy amino acids by changing the growth medium immediately following the induction of DCAFs expression (39). Thus, this approach is a pulse-SILAC variation to measure protein degradation, concomitant with each DCAF temporal expression in a single time point experiment, coupled with MS-based proteomics quantification (Fig. 5*A*). The pulse-chase SILAC experiments were performed on myc-BirA∗-DCAFs and myc-BirA∗ (used as control) expressing U2OS cells. The cells were grown in a medium containing light amino acid (L), and myc-BirA∗-DCAFs and myc-BirA∗ were induced through the addition of doxycycline. We replaced the medium with one containing heavy amino acids (H) and doxycycline. After a 16 h incubation in heavy medium, the cells were lysed and analyzed by MS. However, even though the source of amino acids has been changed, there will remain a small amount of arginine and lysine resulting from recycling of amino acids, following protein degradation or from an enduring intracellular amino acid pool (39). Thus, the amino acid pool available for protein synthesis is composed of a small proportion of residual light amino acid, but mostly heavy ones from the fresh heavy medium. In such an experimental design, the logarithmic ratio of L-intensities (L-DCAFs/L-BirA alone) of most proteins not targeted by DCAFs should be stable overtime. However, for proteins for which the degradation rate increases following the induction of a specific DCAF, we should observe a difference between L-intensities of cells expressing myc-BirA∗ and those expressing myc-BirA∗-DCAF, allowing the identification of proteins targeted by each DCAF (log 2 (L-DCAFs/L-Bira alone) ≤−1, (−log10 *p*-value ≥ 3). For two of the 58 DCAFs (ERCC8 and DDB2), two different times point of incubation in heavy medium were performed (6 h and 16 h, N = 4) to be able to identify (1) proteins that are less abundant in myc-BirA∗-DCAFs expressing cells compared to myc-Bira∗ expressing cells 16 h after switching the light medium to heavy medium and (2) confirm that the abundancy of those selected proteins is decreasing overtime in myc-BirA∗-DCAF expressing cells, comparing two different time points (16 h *versus* 6 h after switching the light medium to heavy medium). Moreover, to define which proteins harbor a decrease in abundancy without any relation with DCAFs expression, we performed a null experiment using two cell lines expressing myc-BirA∗ (Supplemental Fig. S1, *A* and *B*, Supplemental Table S7). Those proteins, referred as background, were removed from the list of potential DCAFs targets (proteins with log 2 (L-BirA cell line 1/L-Bira cell line 2) ≤ −1; (−log10 *p*-value ≥ 3). Twelve and forty proteins were decreased in myc-BirA∗-DDB2 and ERCC8 expressing cells, respectively, compared to control cell line at 16 h time point (Fig. 5, *B* and *D*, Supplemental Table S7). Interestingly, when we compare 16 h to earlier time point (6 h), one protein (TP53BP1) was confirmed to be decreased in myc-BirA∗-DDB2 expressing cells and nine proteins (TMTC3, DHX37, CDC27, KIDINS220, RAB34, MTA3, FLII, UBTF, and NDUFAF3) in myc-BirA∗-ERCC8 (Fig. 5, *C* and *E*). Seven of them have a nuclear or nucleoplasm localization (TP53BP1, DHX37, CDC27, KIDINS220, MTA3, FLII, and UBTF), which correlate the nuclear localization of the CRL4 complex. We also compared proteins decreasing in pulse-SILAC experiment to proteins with a SAINT score over 0.7 in proximity labelling experiments (DCAFbase). None of the protein with a SAINT over 0.7 in myc-BirA∗-DDB2 and ERCC8 proximity labelling experiments were found decreased in pulse-lilac experiments as presented in DCAFbase. We repeated this experiment for the 58 DCAFs (N = 2). The results, presented as Volcano plots, highlight the proteins that had a decreased abundancy, meaning potential increased in their degradation, following the induction of each DCAFs, as indicated by a smaller myc-BirA∗-DCAF/myc-BirA∗ ratio (DCAFbase, Supplemental Table S8). Proteins with a significant (-log10 *p*-value ≥ 3) decrease of L-intensities appear in green and can be sorted with different ratios. To confirm that decrease in abundancy is not due to decrease in gene expression, we performed qPCR on four of the potential targets identified (CDC27, RAB34, TP53BP1 and UBTF, N = 2) (Fig. 5*F*). The expression level of CDC27, RAB34 does not decrease in myc-BirA∗ERCC8 cells compared to myc-BirA∗ expressing cells and two other DCAFs expressing cells (DDB2 and WDTC1). UBTF expression decreases in ERCC8 expressing cells compared to myc-BirA∗ expressing cells, but is similar to that of myc-BirA∗-DDB2 and myc-BirA∗-WDTC1 expressing cells, meaning that this effect on the expression is probably due to DCAF expression no matter which DCAF is overexpressed. We then treated myc-BirA∗-ERCC8 expressing cells with the cullin inhibitor MLN4924 (10 μM during 24 h) to confirm that the degradation observed previously is specific to the activity of cullins. Interestingly, protein abundancy of RAB34 and UBTF was increased after MLN4924 treatment (Fig. 5, *F* and *G* and Supplemental Table S9). DHX37, CDC27, KIDINS220, MTA3, and NDUFAF3 were not detected neither in DMSO- and MLN4924-treated cells. Taking together, these results confirms that UBTF and RAB34 could be targeted for ubiquitination by CRL4^ERCC8^ and then sent for proteasomal degradation. Moreover, this confirms that pulse-SILAC analysis performed in this study is an interesting approach to identify new substrates of DCAFs and CRL4 complex.

### Accessing the Data for all the DCAFs Through DCAFbase

**Fig. 6:**
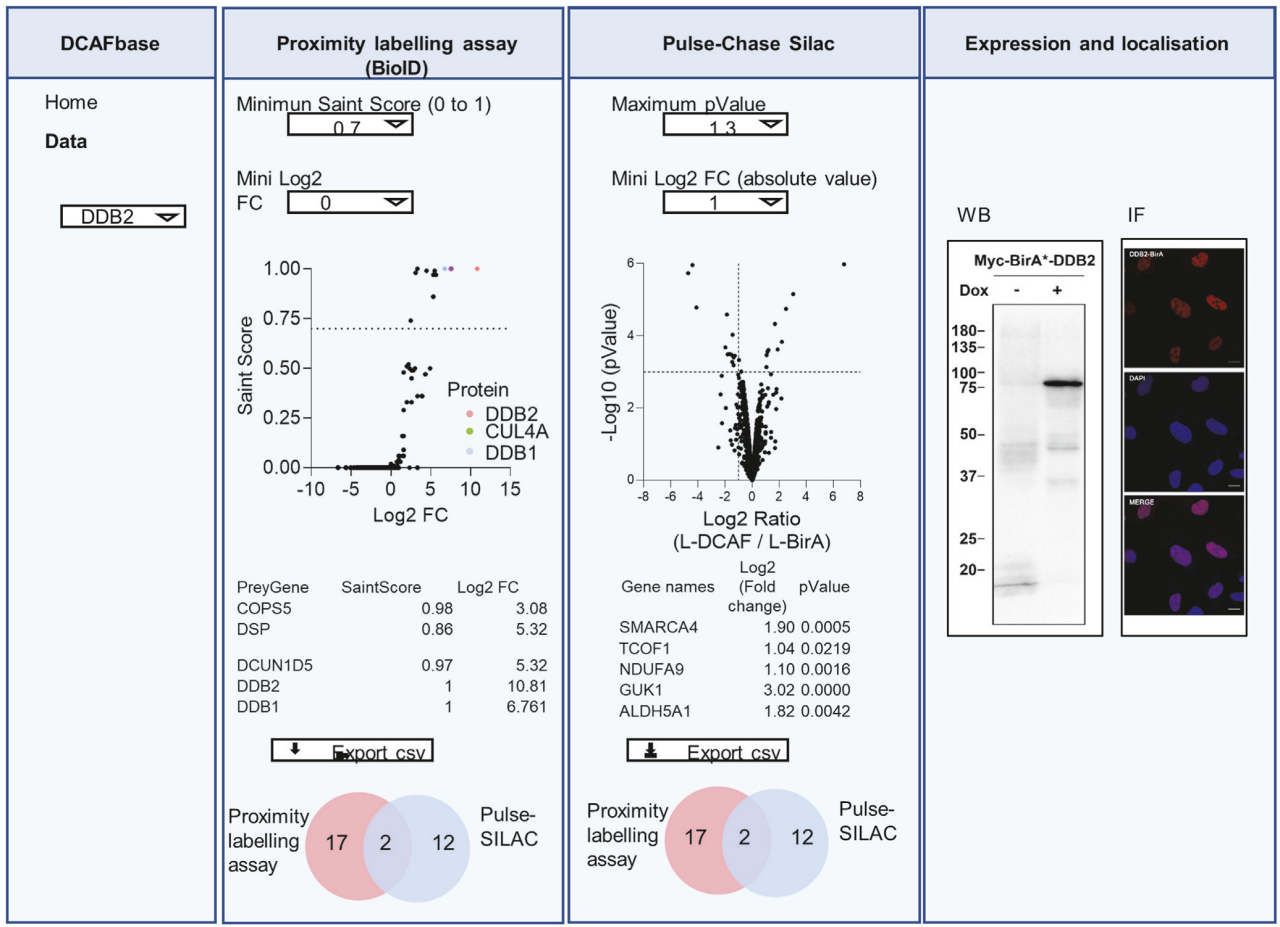
**Accessing the data for all the DCAFs through DCAFbase.** Schematic representing the interface of DCAFbase and data available. Each DCAF can be selected to visualize expression, localization and results from BioID and pulse-SILAC experiments. Results can be sorted according to specific threshold. All data are exportable. DCAF, DDB1-CUL4–associated factor; SILAC, stable isotope labeling with amino acids in cell culture.

Given the massive amount of data generated by the experiments described in this article, which can be tedious to navigate in extensive supplementary tables, we created a simple interface to visualize the data for each DCAF. The online resource, called DCAFbase, allows accessing and visualizing the data for each of the 58 DCAFs in a relational database in a web interface at https://labofmb.github.io/DCAF-web/. The R package is also available at https://github.com/laboFMB/DCAF-web. The interface shows validation for expression and localization (immunoblotting and immunofluorescence microscopy), along with the interactome and degradome data (Fig. 6). These can then be filtered with different score and *p*-value thresholds, with downloadable generated tables. Interactome can be filtered according to the SAINT score and the FC compared to the control condition (cells expressing BirA∗ alone). Degradome data can be visualized as a Volcano plot and filtered according to the FC *p*-value. Moreover, all the graphs are interactive and adapt to table filtering by displaying the selected proteins in a different color, in addition to showing the gene name when a protein of interest is selected in the graph.

## Discussion

Analyses of the expression of DCAF genes in different human tissues revealed variations in DCAF abundancy, with a higher expression in the uterus, ovary, testis, spleen, and thyroid gland, and a generally lower expression in the digestive tract tissues. Specifically, RNA-seq studies on mouse and human tissues showed that the expression of most WD40 genes is very high in testis samples, suggesting an important role in testicular functions (22). Indeed, the CUL4 E3 ubiquitin ligase plays a central role in mammalian spermatogenesis, as shown by male infertility observed in null mutations of the *Cul4A or Cul4B* genes in mice (40, 41). The CRL4 functions appear to be essential in cancer and immortalized cells as well, with approximately 20% of the DCAF showing a high fitness score for essential genes (HeLa, DLD1, HCT116, RPE1, GBM) (33). Also, several studies reported that some DCAFs promote cancer development, and their overexpression is often associated with a poor prognosis (42, 43, 44). For example, even if DTL is almost undetectable in normal human tissues (Fig. 1*A*), it is the most essential DCAF with a BF over 100 in cancer cell lines (Fig. 1*B*). DTL was previously found to play a crucial role in cancer development by degrading the programmed cell death 4 protein, leading to cancer progression (45). Moreover, targeting DTL expression in human hepatocellular carcinoma inhibits cancer cell growth (46). As expected, DDB1 has the highest BF score, meaning it is the most essential gene analyzed and further confirms its central role within the CRL4 complex by bridging the ubiquitin ligase activity with the different adaptors for substrate recognition. However, CUL4A and CUL4B have very low fitness scores, confirming their overlapping functions and compensation of one by the other paralog (Fig. 1*B*), even though they have different localization within the cells. DDB1 was originally identified as a protein involved in the nucleotide excision repair pathway with a high affinity for UV-induced DNA damage sites (47). Moreover, the DDB1–CUL4 ubiquitin ligase complex was recently demonstrated to play a key role in genomic stability, allowing a sister chromatid cohesion during DNA replication (48).

While several DCAFs have been proposed as CRL4 substrate–recognition factors based on the presence of the consensus tandem domain DXXXR/KXWDXR/K (D: Aspartic acid; R/K: Arginine or Lysine; W: Tryptophan), most of them were unconfirmed. To experimentally identify and validate these proteins as associated factors, we first performed extensive proximity labeling assays and DDB1 coimmunoprecipitations, followed by protein identification by MS. Surprisingly, only 14 DCAFs (23%) were enriched in these assays, suggesting that perhaps fewer of these proteins are actually interacting with either DDB1 or Cul4, and that some of them may not be substrate receptors for this complex. This observation is consistent with other large-scale interactomics experiments (BioPlex, BioGRID, and OpenCell), which also identified 13 of these 14 proteins. The BioPlex 3.0 includes two additional DCAFs interacting with DDB1 (DCAF4 and DET1), and more than ten additional DCAFs were found enriched in the BioGRID database, while no additional DCAFs were found in the DDB1 interactome reported in the OpenCell database (37). The BioPlex interactome is an approach similar to ours (large-scale AP-MS) and is very consistent with the DCAFs found associated with DDB1 using our AP-MS or BioID approach. In contrast, the BioGrid database is a compilation of data from a comprehensive curated effort, which also includes data such as cofractionation, two-hybrid, copurification, cocrystal structures, and more. Interestingly, BioPlex 3.0 uses two cell lines (HCT116 and 293T), which could explain the different DCAFs found associated with DDB1 as compared to our study.

Some interactions between DDB1 and some DCAFs might be cell-specific or occur under specific conditions, which is particularly important considering that the expression of the DCAFs is very different in the tissues we examined (Fig. 1*A*). Accordingly, we decided to perform the reciprocal experiment by cloning and expressing all the proposed DCAFs of which we managed to express and validate 58 of the 59 possible DCAFs initially reported. By expressing the DCAFs, we circumvent the possibility that the absence of a DCAF in the DDB1 experiments is due to their absence of expression in the cell line we use. We thus expect that if they can act as substrate receptors for DDB1 and CUL4, we should be able to detect those two proteins in an AP-MS experiment using the DCAFs as baits. In addition, this experiment allowed us to identify the proteins interacting with each of the DCAFs. Once again, only a relatively small fraction of the DCAFs was found interacting with either DDB1 or CUL4, although most of the DCAFs identified in the DDB1 interactome were also found interacting with DDB1 or CUL4 in the reciprocal experiment, confirming their role as a DCAFs. This extensive characterization of the interaction between these DCAFs and DDB1 in cells narrowed down the number of DCAFs. The identification of DCAFs interacting with DDB1/CUL4, and the reciprocal interactome performed, allowed the identification of possible cellular functions for several of these proteins. Indeed, we found some expected functions, such as ubiquitin ligase activity and protein folding, but also extended the known functions of the CRL4 complex into secretory granules and vesicles, nucleic acid binding, and ribosomal translation.

Because the interaction between a ubiquitin–ligase complex and its protein substrates often results in protein degradation, the approach based on protein interactions might not allow the identification of the protein targets of the CRL4 complex. Therefore, using inducible stable cell lines, we decided to identify the proteins that had a decreased abundancy following overexpression of each DCAF. Our hypothesis was that short-term expression of a specific substrate receptor for the DDB1–CUL4 complex would increase ubiquitination of its target proteins, which would increase its degradation by the proteasome. The use of pulse-SILAC labeling is an approach that can measure the difference in turnover of the proteome and allow the quantification following the induction of the individual DCAFs. Interestingly, this approach has allowed us to identify several proteins that were regulated following the expression of each DCAF. For example, we observed that RAB34 and UBTF protein abundancies were decreased, when ERCC8 is expressed and were increased after cullins inhibitor treatment (Fig. 5, *D*, *E* and *G*), suggesting that RAB34 and UBTF are degraded through the CRL complex. Interestingly, both RAB34 and UBTF were recently found to be involved in cancer development. According to TCGA analysis data, RAB34 expression is increased in high-grade glioblastoma compared to lower grades, and UBTF is increased in melanoma compared to normal skin (49, 50). Moreover, we demonstrated that DDB2 overexpression decreases the protein levels of TP53BP1 (Fig. 5, *B* and *C*). Interestingly, TP53BP1 plays a key role in recognition of double strand break DNA damages, particularly through interaction with modified histones including ubiquitination (51, 52, 53). Its negative regulation appears to be important to avoid excessive spreading of TP53BP1 to undamaged chromatin. One way to control accessibility of TP53PB1 to chromatin is to modulate its stability through degradation. Indeed, it was previously demonstrated that TP53BP1 can be degraded directly by proteases, such as cathepsin (54), and/or by ubiquitination involving UbcH7, an E2 ubiquitine ligase, leading to proteasomal degradation under normal and DNA damage condition (55, 56). As such, CRL4^DDB2^ could be part of this cellular control of TP53BP1 abundancy through proteasomal degradation under normal or stress condition. Moreover, DDB2 substrates were previously reported such as several histones (H2S, H3, H4) (27, 28). In our study, these substrates were not found significantly decreased after myc-BirA∗-DDB2 expression. Histones H3.3 was increased (log2 FC = 2.2; *p*-value = 0.07), others were decreased such as HIST1H2AC (log2 FC = −0.5; *p*-value = 0.23), HIST2H2AB (log2FC = −0.32, *p*-value = 0.33), and HIST2H3PS2 (Histone 3, log2 FC = 0.39; *p*-value = 0.57). Interestingly, DDB2 was found to be its own substrate, and it was found decreased over time comparing 6 h *versus* 16 h after low medium replacement (log2 FC = −0.48, *p*-value = 0.11). Previous studies reported that CSB (gene name: ERCC6) is targeted to proteasomal degradation by ERCC8 in a UV-dependent manner (57). CSB was not identified as a target in our study as our cells were not treated with UV. Therefore, we clearly missed CSB, which is a substrate of ERCC8 in a specific context. Moreover, CSB was not detected in our dataset.

Interestingly, none of the proteins modulated in myc-BirA∗ERCC8- and DDB2-expressing cells were identified as interactors in proximity labeling assay. It is possible that DCAFs could harbor a scaffolding role instead of a substrate recognition function (58), which could also explain why most of the interactors found in proximity labeling assay were not identified as potential targets in pulse-SILAC experiments. In such a model, for example, PHIP brings the CRL4 complex to specific DNA marks and switches with RBBP7, leading to degradation of specific substrates such as CDT1 and BUB3 (58, 59). DDB2 is also known to bind DNA on pyrimidine dimers induced by UV exposure, leading to fast degradation of DDB2 and XPC (15, 60). Under UV exposure, DDB2 stimulates the catalytic activity of PARP-1, but is also involved in recruiting the CRL4 complex to DNA damages sites. DDA1 is probably one of the best examples of a DCAF with a structural role of bringing together other proteins in our study. In the proximity labeling experiments, twelve DCAFs were enriched in myc-BirA∗-DDA1–expressing cells, with a SAINT score over 0.7, compared to control cells (DCAF12, DCAF6, DCAF13, DCAF4, VprBP, TRO1AIP2, RBBP7, RBBP4, SNRNP40, PHIP, DTL, WDR5). Interestingly, in myc-BirA∗-DCAF6 proximity labeling experiments, DCAF6 also interacts with DDA1 with a SAINT score of 0.95. Shabek and colleagues speculated that despite its catalytic role, DDA1 interacting with DDB1 can also interact with other DCAFs or DCAFs-bound substrate to facilitate the recruitment of targets to CRL complex or even change the topology of CRL4–substrates complex (61, 62). Taking together, these information indicate that DDA1 can display a scaffolding role with or without switching with them and especially with DCAF6.

In conclusion, our work significantly expands our understanding of DDB1–CUL4–DCAF complex associations, and the reciprocal interaction analyses narrowed down the possible substrate receptors for the DDB1/CUL4 complex with high confidence to seven substrate receptors. Moreover, with pulse-SILAC experiments, we defined the degradomes of each DCAF and identified potential new targets for the CRL4 complex. We were also able to confirm that RAB34 and UBTF are affected by ERCC8, confirming that we could identify potential protein substrates of DCAFs. We collected a large amount of data with the expression, localization, interactome, and degradome of these 58 DCAFs. Therefore, we created an interface allowing easy visualization, where each result is readily accessible for all the DCAFs. The characterization of 58 WD40 proteins called DCAFs gives us a new insight into potential targets of the DDB1–CRL4 E3 ligase complex, uncovering new functions in cells.

## Data Availability

The MS proteomics data have been deposited to the ProteomeXchange Consortium *via* the PRIDE (63) partner repository with the dataset identifiers PXD037482 and PXD042903.

## Supplemental data

This article contains supplemental data.

## Conflict of interest

F.-M. B. is an FRQS Senior scholar (award number 281824). F.-M. B. and P.-É. J. are members of the FRQS-funded “Centre de Recherche du CHUS.” The other authors declare no competing interests.
