# Supplementary figures and images for: Pulse-SILAC and Interactomics Reveal Distinct DDB1-CUL4–Associated Factors, Cellular Functions, and Protein Substrates
Source: Mol Cell Proteomics. 2023 Sep 7;22(10):100644. doi: 10.1016/j.mcpro.2023.100644 (PMC10565876; doi:10.1016/j.mcpro.2023.100644)

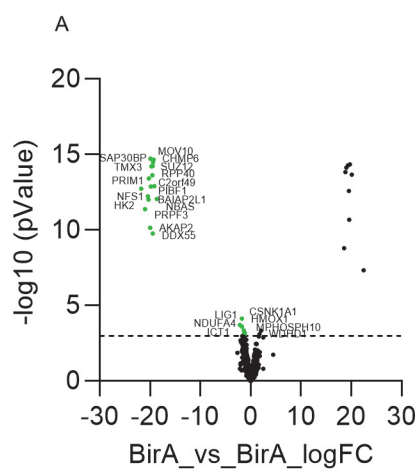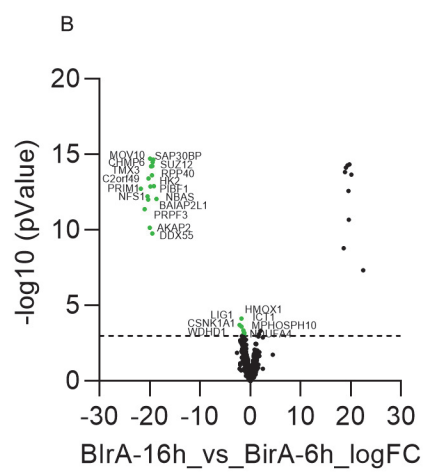

Supplement: Supplemental Figure S1 [file mmc10.pdf]
